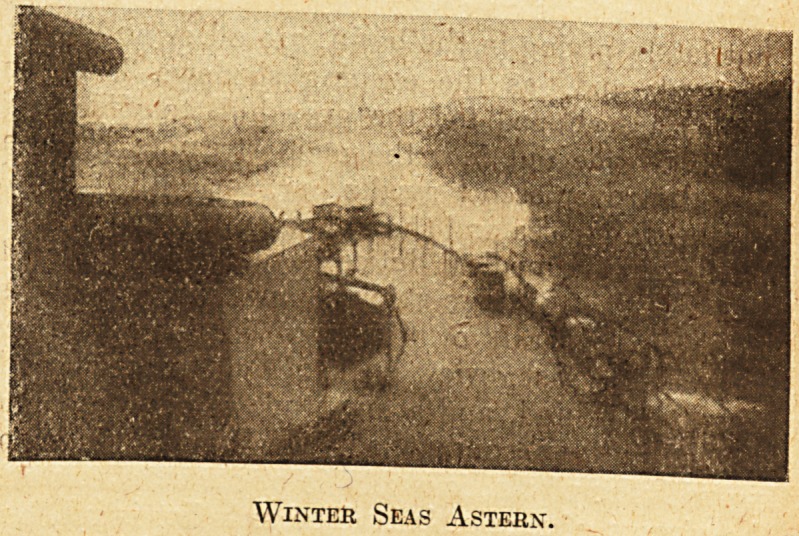# High Sea Wanderings

**Published:** 1919-04-19

**Authors:** 


### NOTES OF A NAVAL SURGEON.

### II. High Sea Wanderings.

A long refit is drawing to a close. Officers and men returning from leave look as though "doc" were the last person whose attention they needed, and the steel mazes of the ship with her varied human complement seem to vibrate with a new vitality. Basin trials have been completed with the bustling assistance of many tugs and now the cruiser lies at the quayside, strained to land by a few stout wires as though impatient at her bonds. Two small gangways throng with an endless human stream which ebbs and flows as the final needs of war are supplied. Stores of all kinds rapidly vanish inboard, ratings on shore duty return, ship's boats, but lately plying in the basin, are all swung in and secured, and, as the evening shadows lengthen, cables are slipped for the grey hull to glide with the flood-tide out into the main stream's breadth. The destroyer escort awaits her, for with nightfall she heads for sea and the unknown future.

Meanwhile medical duties have not been absent. Sick-bay has been re-stocked, medicine chests replenished, a draft of fifty men joining examined for fitness, a last review made of the "sick ashore" to be left behind, and a multitude of odd store and clerical duties accomplished. The hospital, in keeping with the whole, is symbolic of preparedness and the virtues of new paint. The sick list is nil, but once at sea the medico is soon awakened from any dreams that he has nothing to do.

In the later days of the war, the greatest scourge at sea was the now too well-known influenza. The occasion was rare indeed that a ship remained long in contact with land without some one bringing a fresh infection aboard and then, after four days, its ravages would commence. The eight cots and sundry slung hammocks would overflow within twenty-four hours. One mess after another would be cleared and taken over by the sick-berth staff. Izal sprays, blanket-washing, isolation and the rest were of little avail between the close-shut decks. On one occasion, seventy men were down at the same time, and to four of them a farewell was

The Needs of War.

bade at evening quarters when, the flag at half and the white surplice of the chaplain reflected in a streaming, wind-beaten quarter-deck, the ship's company attended service as the bodies were committed to the Atlantic.

Convoy and patrol work has forced many of our

A previous article appeared March 22, page 547.

**Notes of a Naval Surgeon—(*continued*).**

older warships to face harder weather and longer spells than were ever expected by their designers. A low freeboard and the top-heaviness due to a great weight of gun turrets makes rolling tremendous in heavy seas, bringing factors both serious and amusing to bear on a surgeon's work. Should "doc" be a bad sailor himself, one leaves the reader to imagine his woes when called from discreet retirement to scramble to the inaccessible scene of some mishap.

A Sharply Rising Sea.

Green seas sweeping the upper deck frequently smash ships' fittings of all kinds, and it is not surprising that, despite rails and life-lines, human injuries are common. Falls from aloft or down wet hatchway ladders, scalds from upset boiling water, accidents from shifting gear between decks go to swell the rough weather list, for though amazingly inured to such circumstances, the sailor daily runs innumerable risks. Injuries to hands are extremely common. Moving of hoists, guns, hatch-covers, and, in fact, every "gadget" touched in a day's work is accompanied by such weight and resultant force that the slightest slip or lack of vigilance may lead to accident.

Good nursing is not easily procured afloat. Usually a few instances of milder chronic disease are found among the higher and specialised ratings, older men of many years' training, who can ill be spared and with difficulty replaced in an ever-increasing navy. Little more do they need than care and relief from physical strain, but in a war complement with its high percentage of "hostilities only," the inevitable cases of breakdown, of acute rheumatism, and of bronchitis or pneumonia too frequently occur. In fair weather with full ventilation cases are relatively comfortable and the possible treatment satisfactory, but the likelihood of action is always to be borne in mind and evacuation of all unfit men insisted upon whenever the chance occurs. Through one eventful fortnight of the vilest weather the ship both accounted for a Hun surface raider and aided a great merchantman in distress. An acute pneumonia case developed on the fifth day out, and with no hope of a landing the fight for that man's life was waged under every conceivable difficulty. Several times he rallied and was still alive on reaching a sub-tropical port. Hardly was the anchor down before a picket boat was bearing the man's cot through a maze of coral islands to the white low-roofed hospital beyond, and it was with intense disappointment one heard he had reached this palm-shaded refuge only to breathe his last.

Mental derangements and depression are frequently met with after long days afloat. Monotony, strain, and home worries all play their part, and even suicide is not uncommon. There was the instance of a petty officer who, going below to open stores, turned an extremely strong, sharp blade against his own throat and was found some hours later stone dead among the intended flour rations for the day. In the case of a solitary ship, a man overboard is generally a man lost, for none dare stop and search in the possible presence of enemy submersibles. The minimum speed of fifteen knots maintained soon leaves a floating object far astern, and by the time a ship is put about there is, in any case, but poor chance of rescue in the winter seas. All are wisely instructed to wear life-lines when working in positions of danger, and one remembers a man who should be ever thankful. Cleaning guns and knocked off the ship's side by a sharply rising sea, he was towed in mid-ocean for some minutes by his waist-line. Pressure of the rope caused tremendous bruises on his body, and his right shoulder was dislocated, but in sick-bay, after a much-needed gulp of brandy, he was smiling at what would prove a splendid yarn for home consumption.

A wonderful man is the matelot. Either on watch, unrecognisable in duffle suit, pacing hours long in piercing wind and spray, or between times crowded below to play his games or his quaint instruments in an impossibly damp and reeking atmosphere, he remains a marvel of patience and quiet geniality. Such war-time life, above all others, leads to the temptations and excesses ashore, and there one must judge him leniently. The dragging months of sea routine are too little realised by the landsman. The strain is often—in fact always—referred to in every record of the naval war, but the picture is hard to build from imagination alone, and one believes that a few years of lower-deck life in a high seas patrol-ship puts a man to as great a test, both physically and mentally, as he is likely ever to meet in this world.—F. A. K.

Winter Seas Astern.

## Figures and Tables

**Figure f1:**
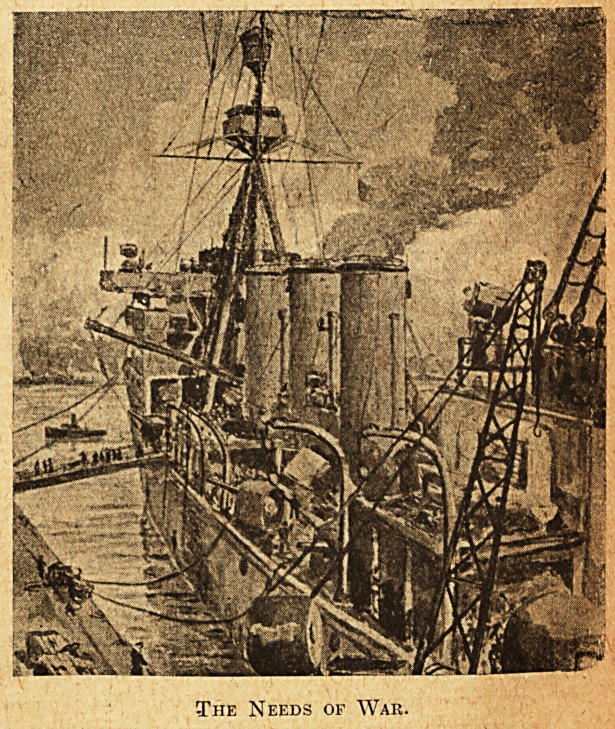


**Figure f2:**
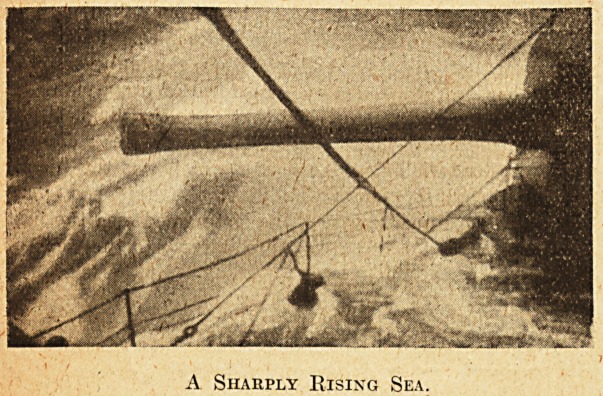


**Figure f3:**